# Serum and Plasma miRNA Expression Levels in Sudden Sensorineural Hearing Loss

**DOI:** 10.3390/ijms26031245

**Published:** 2025-01-31

**Authors:** Desmond A. Nunez, Reyhaneh Abgoon, Printha Wijesinghe, Cathie Garnis

**Affiliations:** 1 Division of Otolaryngology, Department of Surgery, University of British Columbia, Vancouver, BC V5Z 1M9, Canada; 2 Vancouver Coastal Health Research Institute, Vancouver, BC V5Z 1M9, Canada; 3 Division of Otolaryngology-Head Neck Surgery, Vancouver General Hospital, Vancouver, BC V5Z 1M9, Canada; 4 Department of Ophthalmology, Faculty of Medicine, University of British Columbia, Vancouver, BC V5Z 0A6, Canada; 5 Department of Integrative Oncology, British Columbia Cancer Research Centre, Vancouver, BC V5Z 1G1, Canada

**Keywords:** sudden sensorineural hearing loss, microRNA, serum, plasma

## Abstract

Sudden sensorineural hearing loss (SSNHL) is a rapidly developing acquired idiopathic disorder. Differential expressions of microRNAs (miRNAs) have been identified in the acute serum of SSNHL patients. miRNAs are transmitted in both serum and plasma, but it is unknown which better reflects changes associated with inner ear disease. Therefore, we sought to compare the serum and plasma miRNA expression levels in adult SSNHL patients. We extracted and reverse transcribed total RNA from serum and plasma, and analyzed the product with quantitative real-time PCR. hsa-miR-191-5p was used for normalization, and miRNA expression levels were calculated using the delta Ct method. Serum and plasma samples from 17 SSNHL patients (mean age 51.9 years, standard deviation 13.9 years) showed no significant differences in miR-128-3p, miR-132-3p, miR-375-3p, miR-590-5p, miR-30a-3p, miR-140-3p, miR-186-5p, and miR-195-5p expression levels on Wilcoxon signed-rank test analyses. We conclude that plasma and serum are equally suitable for investigating potential miRNA SSNHL disease markers.

## 1. Introduction

Sudden sensorineural hearing loss (SSNHL) is an acquired idiopathic hearing loss that develops within 72 h [1]. Recent studies report an incidence rate of 27–61 cases per 100,000 individuals. These studies also detail age-specific differences in incidence. A peak incidence rate of 77 per 100,000 was reported in the American population aged 65 years and older with an overall slight male predominance [2]. In contrast, Nakashima et al. found a peak incidence of 94 per 100,000 in 60–69 year old patients with the incidence declining thereafter and with an overall female predominance [3].

MicroRNAs (miRNAs) are short RNA chains, typically consisting of 21–23 nucleotides that act as post-transcriptional gene expression regulators. According to current estimates, less than 2000 human miRNAs regulate more than one-third of the cellular transcriptome [4]. MiRNAs play important regulatory roles in most cellular and developmental processes and have been linked to a wide range of human diseases [5,6]. MiRNAs have been shown to modulate up to 60% of protein-encoding genes, influencing the cellular cycle, differentiation, proliferation, and apoptosis [7]. MiRNAs have been found in a variety of body fluids such as serum, plasma, urine, and cerebrospinal fluid and have been shown to be reliable markers of a number of diseases [8]. Many studies have found evidence of miRNA involvement in cancer, coronary heart disease, and neurological disease [9,10,11,12]. Normal inner ear cells express miRNAs, which are important for their development, differentiation, and survival [13,14,15]. Li et al. and Nunez et al. recently found evidence of differentially expressed miRNAs (DEMs) in the blood of SSNHL patients recruited in China and Canada, respectively, compared to local controls [16,17].

The DEMs identified in plasma by comparing 9 SSNHL patients with 3 controls in Li et al.’s study were miR-296/-3667/-15a, miR-1180/-18b/-451a/-24-1/-210/-99b/-190a/-660/-3940/-34a/-1-1/-1-2/-548ay/-95/-1255a/-143/-23a/-548n/-3679/-3074, and miR-4742 [16]. Meanwhile, Nunez et al. identified hsa-miR-590-5p/-186-5p/-195-5p/-140-3p/-128-3p/-132-3p/-375-3p, and -30a-3p as DEMs in serum by comparing 36 SSNHL patients with 12 controls [17].

A number of the above miRNAs have been shown to affect cell metabolism and or survival in different organs. miR-186-5p/-195-5p/ and -590-5p exert control over apoptosis by targeting different genes. The targeting of genes XIAP and CEP55 by miR-186-5p in cardiomyocytes [18] and -195-5p in non-small cell lung cancer cells [19], respectively, triggers apoptosis. MiR-590-5p has also been linked to apoptosis, and inflammation through its targeting of FGF18 in osteoarthritis-related chondrocytes [20].

Cellular metabolic effects have been documented for miR-140-3p through its activation of the phosphatase and tensin homolog (PTEN)/phosphatidyl inositol 3 kinase/protein Kinase B (PI3K/AKT) signaling pathway in bone [21]. In the nervous system, MiR-30a-3p targets SNAP23, which has been linked to the control of synaptic vesicle trafficking and neurotransmitter release, suggesting that it is involved in synaptic plasticity and neuronal function [22].

PI3K/AKT and Rat Sarcoma (RAS) gene signaling pathways are enriched in the target genes of the DEMs identified in SSNHL [17,23]. Additionally, the RAF1 (proto-oncogene, serine/threonine kinase) gene which initiates the Mitogen-Activated Protein Kinase (MAPK) pathway is targeted by miR-15a [16] and -132/-195 [17]. Therefore, SSNHL DEMs likely exert similar metabolic effects to those of miRNAs identified in other organ diseases.

The packaging of miRNAs in various protein complexes or membrane-bound particles, such as exosomes or microvesicles, maintains their stability and shields them from RNase degradation [11,24,25,26]. Scientists have focused on the profile of plasma and serum miRNAs in recent years due to their remarkably high stability across a wide pH range, for extended storage periods including multiple freeze–thaw cycles and resistance to endogenous RNase activity [7]. They are also simple to sample via relatively non-invasive methods, easily detected, and highly disease-specific [27].

Studies of miRNA expression levels in the plasma and serum of healthy individuals arrive at conflicting conclusions with respect to which type of blood sample is ideal. Wang et al. found that the scope and scale of miRNA detection was greater in serum compared to paired plasma samples using Exiqon PCR panels [28]. The same samples when assessed with Taqman cards using 40-cycle threshold values did not show a statistically significant difference in the concentration of miRNAs between serum and plasma samples. However, when the samples were tested with miRNA-specific qPCR Taqman primers, the serum samples demonstrated significantly higher miRNA concentrations than the plasma samples. The pre-amplification step in the Taqman cards process that was not utilized with Exiqon panels or the specific qPCR assays may have obscured the differences in serum and plasma samples. Foye et al. reported the reverse: the scope and scale of miRNA detection was greater in plasma than serum using Nanostring nCounter technology [29]. These contrasting findings support Wang et al.’s conclusion that miRNA expression levels varied with the measurement technology adopted [28]. Inadvertent hemolysis or lysis of other blood cell types during collection can affect both plasma [4] and serum [30] leading similarly to the release of intracellular miRNAs. These released miRNAs can be erroneously interpreted as disease-specific markers. This study investigates if, in SSNHL patients, there are differences between the serum and plasma expression levels of eight miRNAs previously identified to be differentially expressed in SSNHL patients’ serum [17].

## 2. Results

**Figure 1:**
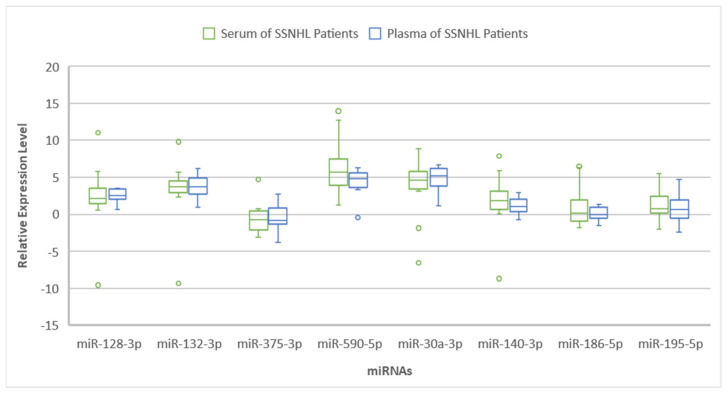
Box and whisker plots showing the relative expression levels of miR-30a-3p/-128-3p/-132-3p/-375-5p/-590-5p/-140-3p/-186-5p, and miR-195-5p in sudden sensorineural hearing loss (SSNHL) patients’ serum and plasma samples. The boxes represent the interquartile range (IQR), with the median marked as a line within each box. The whiskers indicate the minimum and maximum values within 1.5 times the IQR, while outliers are displayed as individual points. Statistical comparisons between serum and plasma miRNA levels were performed using the Wilcoxon signed-rank test.

Paired serum and plasma samples drawn within a median of 3 (inter-quartile range (IQR), 4) weeks of the onset of hearing loss from 8 female and 9 male SSNHL patients with a mean age of 51.9 (±SD, 13.9) years were analyzed (Table 1). One patient had symptoms of dizziness. The mean of the initial pure tone audiometric (PTA)-averaged thresholds across four low (0.5, 1, 2, 3, or 4 kHz) or three high (3, or 4, 6, and 8 kHz) frequencies in the affected ears of the patients was 60.7 (±22.9) dB. This is consistent with moderately severe hearing loss on average in the patients studied [31]. The median and interquartile range (IQR) expression levels of miR-132-3p, -375-3p, -590-5p, -140-3p, -186-5p, -195-5p, 128-3p and -30a-3p were 3.68 (1.59), −0.68 (2.56), 5.71 (3.56), 1.84 (2.45), 0.15 (2.94), 0.79 (2.24), 2.14 (2) and 4.58 (2.40) in serum and 3.76 (2.19), −0.79 (2.17), 4.78 (1.99), 1.1 (1.67), 0.01 (1.46), 0.7 (2.46), 2.54 (1.39), and 5.20 (2.45) in plasma, respectively. There was no statistically significant inter-group difference in the median expression levels of the eight target miRNAs (Figure 1).

**Figure 2:**
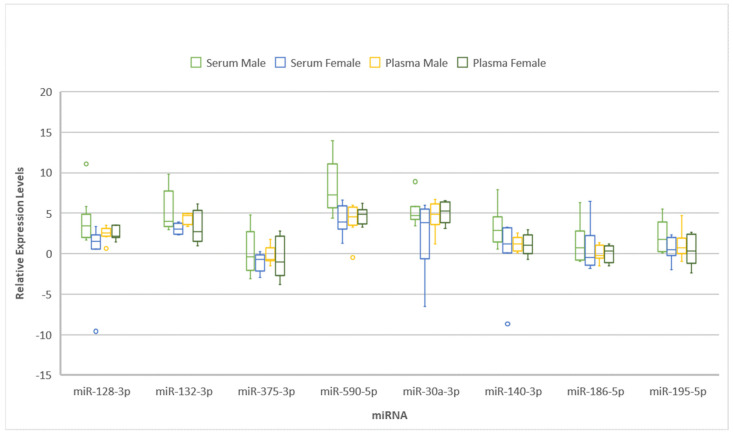
Box and whisker plot showing relative expression levels of miR-30a-3p/-128-3p/-132-3p/-375-5p/-590-5p/-140-3p/-186-5p, and miR-195-5p separately in male and female sudden sensorineural hearing loss (SSNHL) patients’ serum and plasma samples. The box represents the interquartile range (IQR), with the horizontal line inside the box indicating the median. The whiskers extend to the minimum and maximum values within 1.5 times the IQR, and individual points represent outliers beyond this range. Separate plots are provided for serum (green for males and blue for females) and plasma (yellow for males and dark green for females).

The median expression levels of miR-132-3p, -375-3p, -590-5p, -140-3p, -186-5p, -195-5p, 128-3p, and -30a-3p in plasma and serum samples harvested from male and female SSNHL were not statistically different (Figure 2).

**Figure 3:**
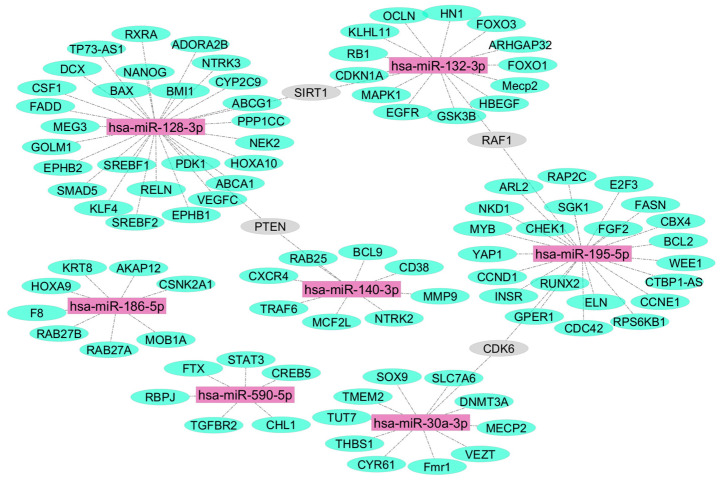
miRNA–target mRNA interactions. The targeting miRNAs are indicated by purple color. mRNAs targeted by a single miRNA are indicated by green color. mRNAs targeted by more than one miRNA are indicated by grey color.

In total, 102 target mRNAs of miR-132-3p, -375-3p, -590-5p, -140-3p, -186-5p, -195-5p, 128-3p, and -30a-3p validated by three strong types of experimental evidence were generated using miRTarBase [32] are illustrated in Figure 3. PTEN, SIRT1, RAF1, and CDK8 mRNAs were targeted by more than one of these seven test miRNAs. No miR-375-5p mRNA targets were identified using the same robust methodology.

**Figure 4:**
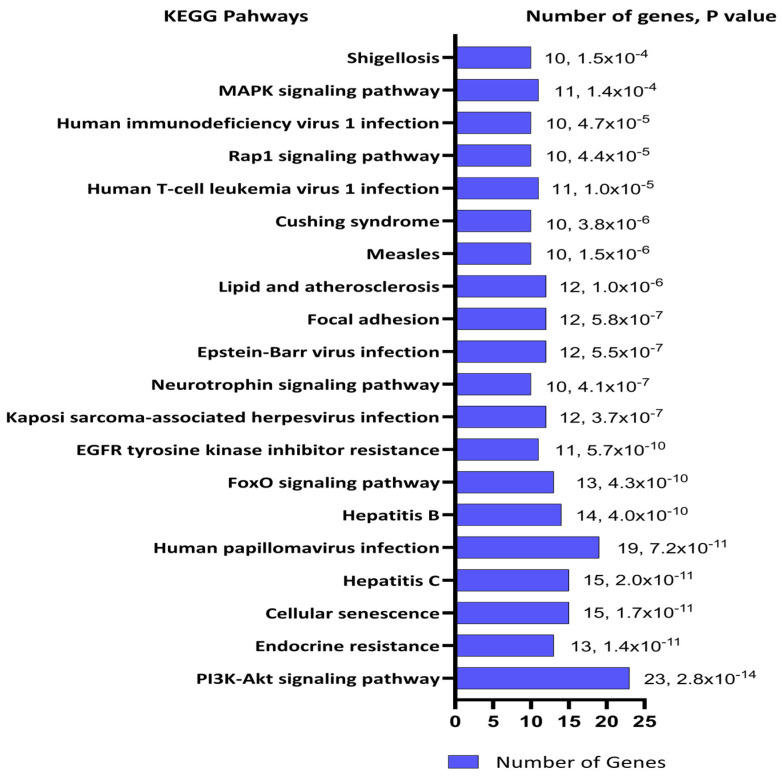
DAVID functional enrichment analysis of target mRNAs or genes in KEGG pathways. The top 20 pathways based on significant *p*-value and gene count excluding pathways involved in cancers are illustrated. The gene count in each pathway is enumerated at the end of each blue pathway bar on the graph.

**Figure 5:**
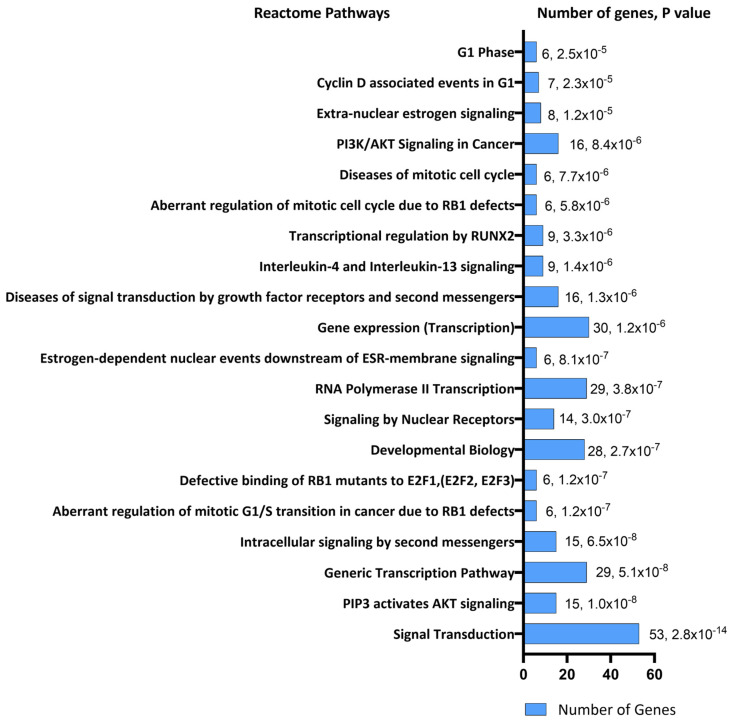
DAVID functional enrichment analysis of target mRNAs or genes in Reactome pathways.

The Database for Annotation, Visualization, and Integrated Discovery (DAVID) was used for functional enrichment pathway analysis of the 102 target mRNAs of the 7 miRNAs identified through miRTarBase resulting in 93 *Homo sapiens genes* [33]. Twenty-three of these genes were identified in the Kyoto Encyclopedia of Genes and Genomes (KEGG) PI3K-Akt signaling pathway representing the highest number of enriched genes in a single KEGG pathway (Figure 4). Signal transduction pathways with 53 target genes were similarly the most enriched Reactome pathways (Figure 5). PI3-Akt, MAPK, RAF, and FOXO genes were common to both KEGG and Reactome enriched pathways.

The top 20 pathways based on significant *p*-values are illustrated. The pathways are listed by significance from *p* = 2.8 × 10^−14^ to 2.5 × 10^−5^. Value labels in the bar chart indicate the number of target genes in each pathway.

## 3. Discussion

Previous investigations into miRNA expression levels in SSNHL studied either plasma [16,34] or serum [17,23] samples exclusively. miR-18b/-23a/-143/-210 were identified as significant DEMs in SSNHL patients’ plasma compared to controls across two studies, albeit with some discrepancies in the findings regarding the direction of expression changes [16,34]. Specifically, Ha et al. reported downregulation of miR-18b and miR-23a, and Li et al. found upregulation of miR-18b and downregulation of miR-23a [16,34]. This disparity might stem from differences in data normalization methods, with Li et al. employing a negative binomial distribution model and Ha et al. using miR-103 or miR-16 as reference miRNAs [16,34]. The serum studies differed from each other in the miRNA populations studied and normalization methods adopted. Zhang et al. investigated exosomal miRNAs using U6 as the reference gene, while Nunez et al. studied circulating miRNAs using the global mean value of 768 target genes [17,23]. These differences likely account for the different range of relevant DEMs quoted in the two serum studies. However, Kyoto Encyclopedia of Genes and Genomes (KEGG) analysis in both studies identified that the target genes of the DEMs were enriched in the phosphatidyl inositol 3 kinase/protein Kinase B (PI3K/Akt) and Ras signaling pathways. Furthermore, KEGG analyses in plasma and serum studies were congruent in postulating MAPK signaling pathway genes as targets of DEMs. Specifically, RAF1 (proto-oncogene, serine/threonine kinase) which initiates the MAPK pathway is targeted by miR-15a [16] and -132/-195 [17]. The expression levels of eight mature DEMs previously identified in SSNHL patients serum by Nunez et al. were studied here [17].

Wang et al. reported higher miRNA expression values in serum compared to paired plasma samples using miRNA-specific qPCR Taqman primers [28]. Conversely, Foye et al. observed an increased scope and scale of miRNA expression in plasma using Nanostring nCounter technology. Additionally, Foye et al. [29] demonstrated that normalizing serum miRNA values against the median of all serum miRNA values effectively reduced the coefficient of variation, while for plasma samples, normalization against the most stable miRNAs proved optimal. In this study, we normalized both serum and plasma values against the value of hsa-miR-191-5p as this miRNA demonstrated consistent expression levels, in both sample types. Thus, the negative value of the expression level of miR-375-3p found here indicates a lower expression level relative to that of miR-191-5p. To understand the relevance of the relative expression levels of miRNAs in SSNHL, attention needs to be directed to comparative studies of SSNHL patients and normal hearing control participants. The expression levels of the eight miRNAs investigated in the current study were not statistically different in paired serum and plasma samples.

Differences in the time elapsed between sample collection and analysis in different studies can introduce variation in total RNA findings. Tsui et al. found that serum and plasma concentrations of mRNA were initially similar when assessed within 4 h of venipuncture [35]. However, over 24 h, while the level in unfiltered plasma stored at 4 °C remained stable, that in serum increased. Serum RNA levels did not, as anticipated, decrease in response to RNase activity and supports the theory that RNAs anneal with DNA making them resistant to both RNase and DNase activity [36] or the RNA is protected in extracellular vesicles or through other protein complexes. This finding also suggests that a greater than 4 h delay in serum RNA analysis will likely result in erroneous miRNA expression level measurements unless some mitigating action, possibly ultra-low temperature storage, is taken. Kim et al. corroborated Tsui et al.’s findings that whole blood holding time even at 4 °C before processing can have dramatic effects on analytical reliability and reproducibility [37]. In the current study, RNA was extracted within one hour of venipuncture in all samples assessed.

Variation in the time of blood sampling relative to the onset of sudden hearing loss may also account for inter-study differences in miRNA findings. However, the 8 miRNAs studied here have been shown to be consistently differentially expressed in serum samples drawn from 1 to 52 weeks after the onset of SSNHL [38]. Therefore, in the current study, miRNA findings in serum and plasma samples collected up to 52 weeks after SSNHL onset are compared. The similarity of the plasma and serum expression levels of the test miRNAs found here suggests that the plasma expression levels of these miRNAs are also stable up to one year after the onset of SSNHL.

Ha et al. [33] reported that pre- and post-treatment plasma miR-15a/-18b/183 and -201 levels correlated with hearing recovery in SSNHL; however, all the patients in their study were treated with intravenous steroids. Spontaneous hearing recovery rates of 65% occur in SSNHL [39] and changes in circulating miRNA levels in untreated patients in relation to hearing recovery have not been reported. There is currently insufficient evidence to determine how much change in circulating miRNAs in SSNHL patients who experience hearing recovery is patient-specific or steroid-induced. In the current study, pairs of serum and plasma samples drawn simultaneously from the same patients were compared. The failure to identify a difference between the levels of the tested miRNAs in serum and plasma samples suggests that any effects of treatment and hearing recovery are equally reflected in both fractions of the patients’ circulating blood.

Studies of haematological, vascular, and cardiac diseases can be anticipated because of their direct relationship to circulating blood to illustrate if serum or plasma samples are superior for the identification of miRNA changes in blood. Mompeón et al. reported that the expression levels of six miRNAs associated with myocardial infarction (MI) were similar in paired plasma and serum samples drawn from control participants without cardiovascular disease CVD [40]. However, significant differences in miRNA expression patterns were observed between serum and plasma samples of CVD patients, with greater variation noted in plasma samples. Both serum and plasma samples of non-ST-elevation myocardial infarction (NSTEMI) patients showed an increase in miR-1 and miR-208b expression, although plasma samples showed greater variation (higher standard deviation) in expression levels. miR-499a expression was significantly increased only in NSTEMI patients’ plasma samples, and the expression of miR-133a and miR-26a was significantly increased and decreased, respectively, only in NSTEMI patients’ serum samples. Interestingly, /-21 displayed a different direction of expression level change in different samples, increasing in serum and decreasing in plasma. This sample-dependant variation in miRNA findings suggests that standardisation of the blood samples plasma or serum utilised for miRNA analysis in CVD is required.

Little is known about the mechanisms that generate the miRNAs found in the circulation of individuals with different diseases, especially diseases of tissues remote from the major blood vessels, or the biological impact of these miRNAs in other parts of the body distant from the primary disease. DAVID functional enrichment analysis utilizing Reactome and KEGG pathways suggests that PI3-Akt, MAPK, RAF, and FOXO genes are likely important targets of the DEMs studied. This agrees with previous KEGG pathway analysis findings on an earlier study of SSNHL patients and controls [17]. The relevance of these circulating miRNAs and their putative targeted genes to the inner ear is supported by functional enrichment pathway analysis of DEMs in differentiated compared to undifferentiated House Ear Institute organ of Corti cells (HEI-OC1). Specifically, the PI3-Akt and MAPK signaling pathways contained the highest number of target genes of the DEMs in differentiated HEI-OC1 cells [15].

Limitations of the current study include the relatively small sample size of 17 patients, and the limited range of miRNAs assessed. The eight miRNAs studied were selected based on our previous study that identified that they were differentially expressed in SSNHL patients’ serum compared to normal non-hearing loss control participants [17]. It is unsafe to extrapolate the study’s finding to other reported SSNHL miRNAs or to miRNAs associated with different disease conditions. Sub-group analysis by sex of miRNA expression levels in serum and plasma samples from the eight female and nine male patients in this study did not identify any statistically significant differences. Caution is also advised concerning the generalizability of these findings to all populations based on this study’s sample of participants drawn from the population of a single North American city. Inter-population genetic variation contributes to differences in disease-specific miRNA findings across studies conducted in different parts of the world [39].

In conclusion, in SSNHL patients, it appears that either serum or plasma samples can be studied to identify select SSNHL-associated miRNA changes, provided RNA is extracted within 60 min of venipuncture. Further, larger sample sized studies are required to confirm these findings and to determine the importance of the eight miRNAs investigated here and others to the progress, treatment, and recovery from SSNHL.

## 4. Materials and Methods

### 4.1. Study Populations and Sampling

In total, 17 adult patients, 18 years and older, presenting with SSNHL as defined by the AAOHNS criteria [41] were recruited at the Department of Otolaryngology, Vancouver General Hospital between 2017 and 2022. All participants’ pure tone audiometric responses were recorded by provincially registered audiologists and/or hearing instrument practitioners. The PTA-averaged thresholds across 4 low (0.5, 1, 2, 3, or 4 kHz) or 3 high (3, or 4, 6, and 8 kHz) as previously described [42], were calculated based on the frequencies demonstrating the greatest degree of hearing loss at presentation. The pure tone audiometric results were utilized to confirm the diagnosis of SSNHL based on the criteria described by Stachler et al. [41] and ascertain that control participants had normal hearing. Specifically, SSNHL participants required audiometrically documented evidence of sensorineural hearing loss of at least 30 dB across three contiguous frequencies.

### 4.2. Clinical Examination and Patient Recruitment

Patients were recruited whether or not they had undergone or were receiving some form of treatment. Otolaryngologist head and neck surgeons or trainees under their supervision registered with the College of Physicians and Surgeons of British Columbia conducted a comprehensive clinical examination of the participants’ ears. Otoscopic examinations of participants ear canals and tympanic membranes were performed to exclude possible causes of hearing loss arising from otitis externa, otitis media, foreign materials, trauma, or other evidence of ear disease. Patients with an identified cause of hearing loss, major medical illness, or coexisting ear pathology were excluded. The study was approved by the University of British Columbia’s Clinical Research Ethics Board and performed in accordance with the relevant guidelines and regulations. Written informed consent was obtained from all participants.

### 4.3. Blood Collection, MicroRNA Extraction, and Reverse Transcription

Blood samples were collected in separate tubes designated for serum and plasma separation within 4 weeks of the onset of the patient’s initial sudden hearing loss, or during subsequent clinical follow-up visits up to 52 weeks after hearing loss onset. Serum and plasma aliquots were stored at −80 °C for RNA extraction. miRNA analysis was undertaken as previously described [38]. In brief, total RNA was extracted from 200 microliters of serum and plasma using miRNeasy Mini Kit (Qiagen, Toronto, ON, Canada) according to the manufacturer’s instructions. Purified RNA was resuspended in 50 microliters of nuclease-free water and stored at −80 °C before reverse transcription (RT) was performed using a TaqMan cDNA synthesis kit (Applied Biosystems, Thermo Fisher Scientific, Waltham, MA, USA), with a preamplification step. The reaction mixture consisted of 2 μL of microRNAs, 3 μL of poly(A) reaction mix, 10 μL of ligation reaction mix, and 15 μL of reverse transcriptase. This was reverse transcribed (RT) at 42 °C for 15 min and deactivated at 85 °C for 5 min. A total of 5 μL of the RT reaction product was incubated with 45 μL of the miR-Amp reaction mix before extracting and diluting cDNA 1:10 with nuclease-free water for miRNA PCR analysis.

### 4.4. MicroRNA Real-Time PCR

Real-time PCR was then performed using Taqman^®^ Advanced miRNA primers and Taqman Fast Advanced master mix on a QuantStudio 3 Real-time PCR System (Applied Biosystems, Thermo Fisher Scientific, Waltham, MA, USA).

Individual qPCR assays were performed three times with a total reaction volume of 20 μL using hsa-miR-30a-3p/-128-3p/-132-3p/-140-3p/-186-5p/-195-5p/-375-3p/-590-5p/ primers (Table 2) and test reference primers hsa-miR-16-5p/-103a-3p/-191-5p according to the manufacturer’s instructions. The PCR thermocycler was programmed for denaturation at 95 °C for 20 s, followed by 40 annealing cycles of 95 °C for 1 s and 60 °C for 20 s. Test miRNA expression levels were calculated for all samples with the delta Ct method using each of the assessed reference primers independently [43]. The reference miRNA that displayed the least variation in delta Ct expression values across the samples was selected as the reference miRNA for all further analyses.

### 4.5. Statistical Methods

Participants’ age, sex, hearing loss side, dizziness if present, and initial pure-tone audiometric (PTA)-averaged thresholds, were summarized with descriptive statistics. Age and initial PTA-averaged thresholds were reported as mean ± standard deviation, while categorical variables such as sex and hearing loss side were summarized as frequencies and percentages. No statistical inter-group tests were performed for age, sex, or dizziness due to the natural paired design of the study, which renders all these factors identical in both groups.

The Kolmogorov–Smirnov (KS) test was first applied to assess the distribution of participants’ ages, PTA-averaged thresholds, and miRNA expression levels for each target miRNA across serum and plasma samples independently. This initial analysis was conducted to determine whether the data followed a normal distribution and to detect any potential outliers. Student’s *t*-tests were used for inter-group analyses if the KS test indicated normally distributed data, and non-parametric Wilcoxon signed-rank tests were employed, if the data did not meet the assumptions of normality. For sex-based sub-group analysis, the Student’s *t*-tests or Wilcoxon signed-rank tests were performed as indicated separately on serum and plasma values for each miRNA to compare male and female patients’ miRNA expression levels. All statistical analyses were performed using SPSS version 26 (IBM, Armonk, NY, USA). Box and whisker plots with means or medians and standard deviations or interquartile ranges were generated with Microsoft Excel.

Putative target mRNAs and genes of the test miRNAs were identified by querying miRTarBase version 9.0 for miRNA–target mRNA interactions supported by all three strong experimental validations such as reporter assay, Western blot, and qPCR [32]. Cytoscape version 3.10.1 [44] was then used to visualize the validated test miRNA–target mRNA interactions. Functional enrichment pathway analysis of the identified target genes was undertaken with KEGG and Reactome pathway tools in The Database for Annotation, Visualization, and Integrated Discovery (DAVID) Bioinformatics (2021 update DAVID Knowledgebase v2024q2) [33]. The top twenty most statistically significant (*p* < 0.05) pathways after Benjamini and Hochberg correction were selected.

## Figures and Tables

**Table 1:** Demographic features of SSNHL patients.

Features	SSNHL Patients
Hearing loss site, n (%) Right ear Left ear	10 (58.8) 7 (41.2)
Initial PTA of the affected ear (mean ± SD in dB)	60.7 ± 22.85
Age in years (mean ± SD)	51.9 ± 13.9
Sex (male: female)	9:8
Dizziness (with dizziness: without dizziness)	1:16

**Table 2:** Test miRNA primer sequences.

miRNA Name	Accession Number MiRBase	Sequence
hsa-miR-128-3p	MIMAT0000424	UCACAGUGAACCGGUCUCUUU
hsa-miR-132-3p	MIMAT0000426	UAACAGUCUACAGCCAUGGUCG
hsa-miR-375-3p	MIMAT0000728	UUUGUUCGUUCGGCUCGCGUGA
hsa-miR-590-5p	MIMAT0003258	GAGCUUAUUCAUAAAAGUGCAG
hsa-miR-30a-3p	MIMAT0000088	CUUUCAGUCGGAUGUUUGCAGC
hsa-miR-140-3p	MIMAT0004597	UACCACAGGGUAGAACCACGG
hsa-miR-186-5p	MIMAT0000456	CAAAGAAUUCUCCUUUUGGGCU
hsa-miR-195-5p	MIMAT0000461	UAGCAGCACAGAAAUAUUGGC
has-miR-191-5p	MIMAT0000440	CAACGGAAUCCCAAAAGCAGCUG
hsa-miR-16-5p	MIMAT0000069	UAGCAGCACGUAAAUAUUGGCG
hsa-miR-103a-3p	MIMAT0000101	AGCAGCAUUGUACAGGGCUAUGA

## Data Availability

The data presented in this study are available upon request from the corresponding author.
